# Treatment patterns and costs for anti-TNFα biologic therapy in patients with psoriatic arthritis

**DOI:** 10.1186/s12891-016-1102-z

**Published:** 2016-06-14

**Authors:** Jacqueline B. Palmer, Yunfeng Li, Vivian Herrera, Minlei Liao, Melody Tran, Zafer E. Ozturk

**Affiliations:** Immunology and Dermatology, Health Economics & Outcomes Research, Novartis Pharmaceuticals Corporation, One Health Plaza, East Hanover, NJ 07936-1080 USA; Outcomes Research Methods & Analytics, US Health Economics & Outcomes Research, Novartis Pharmaceuticals Corporation, East Hanover, NJ 07936-1080 USA; KMK Consulting, Inc, Morristown, NJ 07960-1080 USA; Scott & White Health Plan, Temple, TX/College of Pharmacy, The University of Texas at Austin, Austin, TX 78705 USA; Immunology and Dermatology Medical Affairs Department, Novartis Pharmaceuticals Corporation, East Hanover, NJ 07936-1080 USA

**Keywords:** Anti-TNFα biologic therapy, DMARDs, Treatment patterns, Costs, Switch, Treatment modification

## Abstract

**Background:**

Real-world data regarding anti-tumor necrosis factor alpha (anti-TNFα) biologic therapy use in psoriatic arthritis (PsA) are limited; therefore, we described treatment patterns and costs of anti-TNFα therapy in PsA patients in the United States.

**Methods:**

PsA patients (*N* = 990) aged ≥18 years who initiated anti-TNFα therapy were selected from MarketScan claims databases (10/1/2009 to 9/30/2010). Number of patients on first- (*n* = 881), second- (*n* = 72), or third- or greater (*n* = 37) line of anti-TNFα therapy, persistence, time-to-switch or modification, pharmacy and medical costs (measured per patient per month [PPPM]) for each line of therapy were observed during the 3-year follow-up.

**Results:**

PsA patients receiving only one line of anti-TNFα therapy remained on first-line for ~17 months while those who switched to second- or third- or greater persisted on first-line for ~11 to 12 months, respectively. Time to first-line modification was longer for patients who switched to third- or greater line therapy (7 months) than those who did not switch or switched to second-line (range, ~2 to 4 months). Time-to-switch and time to first-line modification was progressively shorter with each line of therapy for patients who received third- or greater line. PPPM medical costs were higher for patients who did not switch ($322) than those who switched to second- ($167) or third- or greater ($217) line. PPPM pharmacy costs were greater for patients with third- or greater line therapy ($2539) than those who did not switch ($1985) or switched to second-line ($2045).

**Conclusion:**

While the majority of patients received only one line of anti-TNFα therapy, a subset of patients switched to multiple lines of therapy during the 3-year follow-up period. Persistence and therapy modifications differed between these patients and those receiving only one line. Overall medical costs were highest for patients who did not switch, and pharmacy costs increased as patients switched to each new line of therapy.

## Background

Psoriatic arthritis (PsA), a type of the spondyloarthritis [1–4], is a chronic autoimmune disorder that affects the peripheral and axial joints, nails, entheses, and is commonly associated with psoriatic skin lesions [5, 6]. Although symptoms can vary, patients with PsA often suffer from joint pain, stiffness, swelling, dactylitis, nail psoriasis, and fatigue [6]. In addition, patients in the later stages of disease may experience osteolysis with destruction of the joint cartilages and boney surfaces, potentially resulting in severe deformities [7]. In the United States (US), approximately 30 % of patients with psoriasis develop PsA, with the PsA prevalence estimated between 0.10 and 0.25 % of the overall population [6, 8]. PsA is associated with a number of comorbidities, including hypertension, cardiovascular disease, obesity, depression, and anxiety, and is linked to a decrease in the quality of life [6, 9–12]. Direct annual healthcare costs related to PsA were estimated to be as high as $1.9 billion in 2012 [6]. Total indirect healthcare costs accounted for 52 to 75 % of total healthcare costs, with both direct and indirect costs reported to increase with disease severity [6].

The American Academy of Dermatology (AAD) and The European League Against Rheumatism (EULAR) treatment guidelines have suggested a stepwise approach for treating PsA based on symptoms, disease severity, joint involvement, and extent of inflammation [13–17]. Mild disease is typically treated with nonsteroidal anti-inflammatory drugs (NSAIDs) or intra-articular corticosteroid injections [13, 17]. If inflammation is persistent, the guidelines suggest using oral nonbiologic disease-modifying antirheumatic drugs (DMARDs), such as methotrexate (MTX) [13, 17]. If traditional DMARDs are unable to control the signs and symptoms of PsA, the use of biologics is recommended [13, 17]. Biologics approved and currently available for treating moderate-to-severe PsA include the anti-tumor necrosis factor α (anti-TNFα) drugs adalimumab, etanercept, golimumab, infliximab, certolizumab pegol; and the interleukin 12 (IL-12) and interleukin 23 (IL-23) inhibitor ustekinumab [18–23]. These agents have been reported in numerous clinical studies as effective in managing symptoms such as dactylitis, enthesitis, and spondylitis, as well as skin and nail disease [13, 14, 17, 24–28]. Recently updated guidelines from the Group for Research and Assessment of Psoriasis and Psoriatic Arthritis (GRAPPA) have recommended specific treatments based on clinical domains of disease activity (i.e., peripheral arthritis, axial disease, enthesitis, dactylitis, skin, or nails) [25, 26, 29, 30].

Current treatment guidelines offer no guidance in terms of what sequence of anti-TNFα biologic therapy should be used. Patients often start with an anti-TNFα biologic therapy (i.e., first-line therapy) and switch to another line of therapy (i.e., second-line and third-line etc.) due to lack of effectiveness, tolerability or safety [13, 17, 25, 26, 29, 30]. If anti-TNFα biologic therapy is lacking efficacy, current treatment guidelines suggest the addition or modification of nonbiologic therapy (e.g., changing dose or adding or removing a DMARD) [13, 17, 25, 26, 29, 30].

To date, no prior US studies have stratified administrative claims data by lines of anti-TNFα biologic therapy [31–35]; therefore, little is known regarding how often a patient with PsA switches from one anti-TNFα biologic therapy to another (e.g., first anti-TNFα biologic [first-line] to second anti-TNFα biologic [second-line]), and whether a connection exists between treatment modification and switching. In addition, multiple factors may affect medical and drug costs such as functional disability, disease severity, treatment response, dosing schedule, and switching or modifying therapy [6, 24, 27, 34, 36, 37]. Although several studies have reported the annual direct costs of anti-TNFα biologic therapy (i.e., drug and administration costs) for PsA treatment [34, 35, 38, 39], none have investigated the relationship between healthcare costs and switching of anti-TNFα biologic therapy in patients with PsA. Previous US claims database analyses have assessed the treatment patterns and healthcare costs of patients with PsA who received anti-TNFα biologic therapy over a 1-year period [31–35, 38–40]. These studies evaluated the frequency and duration of anti-TNFα biologic therapy (i.e., persistence, discontinuation, restarting and/or switching anti-TNFα biologic therapy), and the addition of another medication to the anti-TNFα biologic therapy [31–35]. Although these claims studies reported treatment patterns and healthcare costs over a 1-year follow-up period, it is unclear whether these findings are maintained over more extended periods of time. Since PsA is a chronic disease, with patients typically on therapy for multiple years, the purpose of this study was to observe the treatment patterns and healthcare costs associated with anti-TNFα biologic therapy over a 3-year follow-up period in patients with moderate-to-severe PsA identified from US claims databases.

## Methods

### Data source

This study utilized a retrospective observational claims database in the US known as Truven Health Analytics MarketScan® Research Databases (the Commercial Claims and Encounters Database [Commercial] and the Medicare Supplemental and Coordination of Benefits Database [Medicare] from January 1, 2005 to September 30, 2013. The Commercial Claims and Encounter Database contains the healthcare experience of individuals who are active employees and early retirees, and includes coverage under fee-for-service (FFS), point of-service (POS), and health maintenance organizations (HMOs) [41–43], including de-identified medical claims (inpatient, outpatient, and emergency room [ER]) and pharmacy claims linked to plan enrollment information. The Medicare database consists of the healthcare information of retirees with Medicare supplemental insurance paid by the employee, any out-of-pocket patient expenses, and portion of the payment [42, 43]. These databases have patient information relatd to demographics, healthcare utilization, comprehensive prescription drug information, and payment costs, and inpatient and outpatient detailed cost, use, and outcomes data [42, 43].

All study data were accessed using techniques compliant with the Health Insurance Portability and Accountability Act of 1996, and no identifiable or protected health information was extracted during the course of the study, hence, the study did not require informed consent or institutional review board approval. Data are not to be shared due to the proprietary nature [42, 43].

### Sample selection and patient population

The treatment identification period was from October 1, 2009 to September 30, 2010. The study population (aged ≥18 years) was selected from commercial and Medicare health plan members identified with ≥1 claim for an anti-TNFα biologic therapy of interest during the treatment identification period and with ≥1 non-rule-out International Classification of Diseases, Ninth Revision, Clinical Modification (ICD-9-CM) claim for PsA (ICD-9-CM code 696.0) before the index date and after January 1, 2005. The index (initiation) date was defined as the first observed claim for use of an anti-TNFα biologic therapy. Patients had to be continuously enrolled with medical and pharmacy benefits 6 months prior to the index date and through the 3-year follow-up period. The follow-up period was defined as the 3 years that followed the index date. Patients with an ICD-9-CM code recorded in claims during the 6-month baseline period and the 3-year follow-up period for rheumatoid arthritis (RA) (714.x) and ankylosing spondylitis (AS) (720.0) were excluded. Patients were also excluded if they used an anti-TNFα biologic therapy within 6 months prior to the index date (to include only new anti-TNFα biologic users).

### Demographic and baseline patient characteristic variables

Demographic continuous and categorical variables were age, gender, geographic region (Northeast, North central, South, West, Unknown), and insurance type (HMO and POS capitation, FFS], Unknown). Clinical categorical variables were comorbidities and first-line anti-TNFα biologic therapy. Comorbidities of interest included hypertension (ICD-9-CM: 362.11, 401.0–405.0, 437.2), hyperlipidemia (ICD-9-CM: 272.0–272.4), type 2 diabetes (ICD-9-CM: 249.0, 250.0, 357.2, 362.0, 366.41), and ischemic heart disease (ICD-9-CM: 410.0–414.0, 414.12, 414.2, 414.3, 414.8, and 414.9).

### Outcome measures

The data set and outcome measures evaluated and methodology used in this study are similar to another recently published study that focused on treatment patterns and healthcare costs of patients with AS in the United States over a 3-year follow-up period [44].

The current study had four key outcomes of interest associated with anti-TNFα biologic therapy: number of patients per line of therapy; duration of treatment; time to treatment modification and healthcare resource costs associated with line of treatment (medical and pharmacy costs). For the first outcome, the number of patients on each “n” th-line of therapy (first-, second- or third) was reported. Treatment duration was measured as persistent use of an anti-TNFα biologic (defined as time from initiation of the treatment line to discontinuation [e.g., a gap in treatment of >60 days]) or as time to switch to the next treatment line, or whichever came first. Time to first treatment modification was defined as the time from initiation of an anti-TNFα treatment to the first modification of that treatment. Treatment modifications included an increase or decrease in dose of an anti-TNFα biologic or DMARD, or an add-on, removal, or change of a DMARD. Healthcare resource costs were reported as medical costs (hospitalizations, office visits, ER visits) and pharmacy drug costs (anti-TNFα therapy and DMARDs) per patient per month (PPPM). Anti-TNFα therapies of interest were etanercept [19], adalimumab [18], infliximab [21], and golimumab [20]. Only anti-TNFα therapies approved during the study period were included. Non-biologic DMARDs included azathioprine [45], hydroxychloroquine sulfate [46], leflunomide [47], sulfasalazine [48], cyclosporine [49], methotrexate [50], and the phosphodiesterase 4 inhibitor, apremilast [51].

### Statistical analysis

All data were analyzed descriptively. Patient-level analyses included demographics, number of patients on one or more lines of anti-TNFα biologic therapy, number of patients initiating each anti-TNFα agent of interest, and number of patients who switched treatments (any switch or ≥ one switch). Subgroup analyses reported the mean (standard deviation [SD]) duration (days) patients persisted on each line of treatment, time to switch to the next line of treatment, time to the first treatment modification, and time from first treatment modification to switch. Medical costs were hospitalizations, office, and ER visits. Pharmacy drug costs included the cost of anti-TNFα biologic and DMARD treatment. The value for each medical and pharmacy outcome was calculated as total PPPM cost incurred from initiation of an anti-TNFα biologic treatment to discontinuation of treatment or end of the 3-year follow-up period (whichever came first) divided by the number of covered members per months from initiation to treatment discontinuation or end of the follow-up period (whichever came first).

## Results

### Demographics and baseline characteristics

**Fig. 1:**
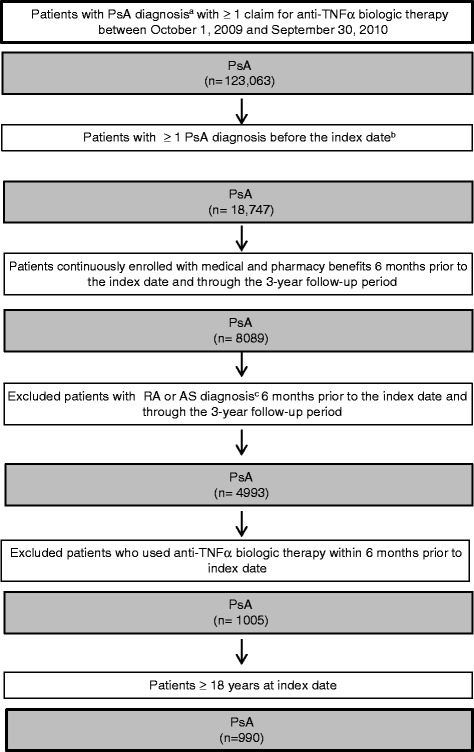
Patient selection flowchart. ^a^The index date is the date of the use of the first anti-TNFα biologic. ^b^A diagnosis of PsA was established according to the ICD-9-CM code 696.0. ^c^The ICD-9-CM codes for RA (ICD-9-CM code: 714.x) or AS (ICD-9-CM code: 720.0) were used for diagnosis. AS, ankylosing spondylitis; ICD-9-CM, International Classification of Diseases, Ninth Revision, Clinical Modification; PsA, psoriatic arthritis; RA, rheumatoid arthritis; TNFα, tumor necrosis factor-α

The final study population included 990 PsA patients who started anti-TNFα biologic therapy between October 1, 2009 and September 30, 2010 (Fig. 1). At baseline (index date), the mean (SD) average age of the study population was 49.0 (10.9) years and was comparable across the lines of therapy (Table 1). A lower percentage of females received only one line of anti-TNFα biologic therapy (43.9 %) compared with those who received a second- (58.3 %) or third- or greater (59.5 %) line of therapy. A higher percentage of patients with PsA in each line of therapy were from the Southern region of the United States compared with other regions, and most (81.5 %) had FFS health insurance (Table 1). The most common first-line anti-TNFα biologic therapies used were etanercept (43.9 %) and adalimumab (44.1 %). Over the 3-year follow-up, 63.5 % of patients with PsA had at least one comorbidity—the most frequently reported were hypertension (45.6 %), hyperlipidemia (37.9 %), and type 2 diabetes (22.6 %) (Table 1).

**Table 1:** Summary of demographics and baseline characteristics

	Patients With Only First-line Biologic Therapy (*n* = 881)^a^	Patients With Second-line Biologic Therapy (*n* = 72)^b^	Patients With Third-line or Greater Biologic Therapy (*n* = 37)^a,b^	Overall (*N* = 990)
Age (y), mean (SD)	49.1 (11.0)	48 (10.9)	48.8 (9.5)	49.0 (10.9)
Female, n (%)	387 (43.9)	42 (58.3)	22 (59.5)	451 (45.6)
US region, n (%)				
Northeast	127 (14.4)	11 (15.3)	4 (10.8)	142 (14.3)
North central	209 (23.7)	11 (15.3)	8 (21.6)	228 (23.0)
South	357 (40.5)	32 (44.4)	15 (40.5)	404 (40.8)
West	182 (20.7)	18 (25.0)	10 (27.0)	210 (21.2)
Unknown	6 (0.7)	0	0	6.0 (0.6)
Health insurance, n (%)				
FFS	721 (81.8)	54 (75.0)	32 (86.5)	807 (81.5)
HMO and POS capitation	137 (15.6)	18 (25.0)	5 (13.5)	160 (16.2)
Missing/unknown	23 (2.6)	0	0	23 (2.3)
Index biologic therapies, n (%)				
Etanercept	389 (44.2)	31 (43.1)	15 (40.5)	435 (43.9)
Adalimumab	391 (44.4)	32 (44.4)	14 (37.8)	437 (44.1)
Infliximab	67 (7.6)	3 (4.2)	7 (18.9)	77 (7.8)
Golimumab	34 (3.9)	6 (8.3)	1 (2.7)	41 (4.1)
Comorbidities, n (%)^c^				
Type 2 diabetes	203 (23.0)	11 (15.3)	10 (27.0)	224 (22.6)
Hypertension	400 (45.4)	33 (45.8)	18 (48.6)	451 (45.6)
Hyperlipidemia	337 (38.3)	23 (31.9)	15 (40.5)	375 (37.9)
Ischemic heart disease	75 (8.5)	4 (5.6)	4 (10.8)	83 (8.4)
Any of the above	563 (63.9)	41 (56.9)	25 (67.6)	629 (63.5)

*PsA*, psoriatic arthritis; *FFS*, fee-for-service; *HMO*, health maintenance organization; *POS*, point-of-service; *SD*, standard deviation; *TNFα*, tumor necrosis factor-α

^a^Biologic therapy refers to the following anti-TNFα agents: etanercept, adalimumab, infliximab, and golimumab. ^b^Among this group of patients, 18 (43 %) switched to a fourth-line of biologic treatment. ^c^Identification was based on non–rule-out diagnoses

### Persistent use and time to switch of anti-TNFα therapy

Over the 3-year follow-up, PsA patients receiving only one line of anti-TNFα biologic therapy persisted on their therapy for 17.4 months (mean [SD]; 522.3 [418.9] days) while those who switched to a second- or third- or greater line therapy persisted on first-line therapy for 11.6 months (348.5 [308.7] days) or 10.8 months (325.2 [239.9] days), respectively (Table 2). Time to first treatment modification for first-line therapy was shortest for patients who received two lines of anti-TNFα biologic therapy (mean [SD]; 58.4 [102.7] days or 1.9 months), followed by those receiving one line of therapy (119.4 [208.7] days or 4 months) and those receiving three or more lines of therapy (219.8 [295.4] days or 7.3 months). In patients who received three or more lines of therapy during the 3-year follow-up period, time to switch and time to first treatment modification were progressively shorter with each new line of therapy. Time from first modification of therapy to treatment switch was longer for patients who switched to a second line of therapy (mean [SD]; 385.3 days [277.1] or 12.8 months) compared with those who switched to a third- or greater line of therapy (143.5 [199.0] days or 4.8 months) (Table 2).

**Table 2:** Treatment patterns for anti-TNFα biologic therapy in patients with PsA for the 3-year follow-up

	Patients With Only First-line Anti-TNFα Biologic Therapy (*n* = 881)^a^	Patients With Second-line Anti-TNFα Biologic Therapy (*n* = 72)^a^	Patients with Third-line or Greater of Anti-TNFα Biologic Therapy (*n* = 37)^a^
Persistent use of anti-TNFα biologic therapy (days), mean (SD)^b^			
First-line	522.3 (418.9)	348.5 (308.7)	325.2 (239.9)
Second-line	N/A	447.3 (327.6)	126.8 (197.9)
Third-line or greater	N/A	N/A	251.6 (305.2)
Time to switch anti-TNFα biologic therapy(days), mean (SD)^c^			
First-line	N/A	349.5 (308.7)	326.2 (239.9)
Second-line	N/A	N/A	127.8 (197.9)
Third-line or greater	N/A	N/A	40.2 (49.8)
Time to first modification of anti-TNFα biologic therapy (days), mean (SD)^d,e^			
First-line	119.4 (208.7)	58.4 (102.7)	219.8 (295.4)
Second-line	N/A	189.5 (226.0)	76.1 (238.9)
Third-line or greater	N/A	N/A	24.3 (33.3)
Time from first modification of anti-TNFα biologic therapy to switch (days), mean (SD)^f^			
First-line	N/A	385.3 (277.1)	143.5 (199.0)
Second-line	N/A	N/A	38.1 (53.7)
Third-line or greater	N/A	N/A	42.0 (65.6)

*PsA* psoriatic arthritis, *N/A* not available, *SD* standard deviation, *TNFα* tumor necrosis factor-α

^a^Anti-TNFα biologic therapy refers to the following anti-TNFα agents: etanercept, adalimumab, infliximab, and golimumab

^b^Persistent use was defined as time from initiation of the line of treatment to discontinuation (a gap in treatment of >60 days) of the line of treatment or switch to the next line of treatment (whichever came first)

^c^Time to switch is defined as time from initiation of the line of anti-TNFα biologic treatment to switch to the next line of anti-TNFα biologic treatment

^d^Time to first modification of anti-TNFα biologic therapy was defined as time from initiation of the line of anti-TNFα biologic treatment to first modification on that line of treatment

^e^Modification of anti-TNFα biologic therapy included: biologic dose increase or dose decrease, DMARD added, changed, removed, or DMARD dose increase/decrease; Benchmark DMARD to identify first-line DMARD change: most recent DMARD in 60-day prior to index biologics; Benchmark DMARD to identify second-/third-line DMARD change: most recent DMARD in the previous line

^f^Time from first modification of anti-TNFα biologic therapy to switch was defined as time from first modification on the line of anti-TNFα biologic treatment to switch to the next line of treatment

### Treatment modification of anti-TNFα biologic therapy

Modification of first-line anti-TNFα biologic treatment occurred in 21.1 % of patients across all lines of therapy over the follow-up period. Patients who did not switch had fewer first-line treatment modifications (19.8 %) compared with patients who switched to a second- (31.9 %) or third- or greater (32.4 %) line of therapy during the follow-up period (Table 3). In all patients, the most common modifications to first-line anti-TNFα biologic therapy were the addition or removal of a DMARD, and change to another DMARD (Table 3). During their second-line of therapy, patients who received only two lines of therapy often added a DMARD (13.9 %), while those who received at least three or more lines of therapy commonly discontinued a DMARD (21.6 %). During their third-line or greater of therapy, the addition of a DMARD (21.6 %) or removal of a DMARD (10.8 %) were the most common treatment modifications for patients with three or more lines of therapy. Changes in dose of anti-TNFα biologic or DMARD therapy were uncommon (≤2.8 %) across the all lines of therapy for both patients who did and did not switch.

**Table 3:** Summary of treatment modification for anti-TNFα biologic therapy in patients with PsA for the 3-year follow-up

	Patients With Only First-line Anti-TNFα Biologic Therapy (*n* = 881)^a^	Patients With Second-line Anti-TNFα Biologic Therapy (*n* = 72)^a^	Patients with Third-line or Greater of Anti-TNFα Biologic Therapy (*n* = 37)^a^
First-line, n (%)			
Anti-TNFα increase	6 (0.7)	0	0
Anti-TNFα decrease	7 (0.8)	0	1 (2.7)
DMARD add-on	74 (8.4)	8 (11.1)	7 (18.9)
DMARD removal	59 (6.7)	12 (16.7)	2 (5.4)
DMARD drug change^b^	27 (3.1)	2 (2.8)	2 (5.4)
DMARD dose increase	2 (0.2)	1 (1.4)	0
DMARD dose decrease	2 (0.2)	0	0
Any of the above	177 (19.8)	23 (31.9)	12 (32.4)
Second-line, n (%)			
Anti-TNFα increase	N/A	2 (2.8)	0
Anti-TNFα decrease	N/A	2 (2.8)	0
DMARD add-on	N/A	10 (13.9)	0
DMARD removal	N/A	4 (5.6)	8 (21.6)
DMARD drug change^b^	N/A	3 (4.2)	2 (5.4)
DMARD dose increase	N/A	0	0
DMARD dose decrease	N/A	0	0
Any of the above	N/A	21 (29.2)	10 (27.0)
Third-line or greater, n (%)			
Anti-TNFα increase	N/A	N/A	0
Anti-TNFα decrease	N/A	N/A	0
DMARD add-on	N/A	N/A	8 (21.6)
DMARD removal	N/A	N/A	4 (10.8)
DMARD change^b^	N/A	N/A	0
DMARD dose increase	N/A	N/A	0
DMARD dose decrease	N/A	N/A	0
Any of the above	N/A	N/A	12 (32.4)

*DMARD* disease-modifying antirheumatic drug, *TNFα* tumor necrosis factor-α

^a^Anti-TNFα biologic therapy refers to the following anti-TNFα agents: etanercept, adalimumab, infliximab, and golimumab

^b^First-line DMARD change was the most recent DMARD in 60-day prior to index anti-TNFα biologic treatment and second- and third-line or greater DMARD change was the most recent DMARD in the previous line

### Medical and pharmacy costs

PPPM medical costs were less than PPPM pharmacy costs across all lines of treatment over the 3-year follow-up period (Table 4). PPPM medical costs were greater for patients who did not switch (mean [SD]; $322 [$1854]) than for those who switched to a second- ($167 [$363]) or third- or greater ($217 [$86]) line of anti-TNFα biologic therapy. In patients who switched to three or more lines of therapy, the PPPM medical costs of the first-line therapy were higher ($282 [$595]) than the second- ($79 [$99]) or third- or greater ($107 [$88]) line of therapy. Overall, PPPM pharmacy costs were higher for patients with three of more lines of anti-TNFα biologic therapy (mean [SD]; $2539 [$1115]) compared with those who did not switch therapy ($1985 [$833]) or switched to a second-line of therapy ($2045 [$650]). Switching to a second-line of anti-TNFα biologic therapy was associated with an increase in pharmacy costs. In the group of PsA patients who received three or more lines of anti-TNFα biologic therapy, PPPM pharmacy costs for the third- or greater line of therapy were lower (mean [SD]; $2126 [$2551]) than the first- ($2515 [$1800]) and second-line ($2947 [$1927]) therapies.

**Table 4:** Mean medical and pharmacy cost PPPM of PsA patients receiving anti-TNFα therapy for the 3-year follow-up^ab^

	Patients With Only First-line Anti-TNFα Biologic Therapy (*n* = 881)^c^	Patients With Second-line Anti-TNFα Biologic Therapy (*n* = 72)^c^	Patients With Third-line or Greater Anti-TNFα Biologic Therapy (*n* = 37)^c^
Medical Cost PPPM, Mean (SD)^d^			
First-line	$322 ($1854)	$127 ($247)	$282 ($595)
Second-line	N/A	$156 ($291)	$79 ($99)
Third-line or greater	N/A	N/A	$107 ($88.0)
Overall	$322 ($1854)	$167 ($363)	$217 ($86.0)
Pharmacy Cost PPPM Mean (SD)^e^			
First-line	$1985 ($833)	$2082 ($836)	$2515 ($1800)
Second-line	N/A	$2114 ($778)	$2947 ($1927)
Third-line or greater	N/A	N/A	$2126 ($2551)
Overall	$1985 ($833)	$2045 ($650)	$2539 ($1115)

^a^The value for each of the medical and pharmacy costs was calculated as total PPPM cost incurred from initiation of an anti-TNFα biologic treatment to discontinuation of treatment or end of the 3-year follow-up period (whichever came first) divided by the number of covered members per months from initiation to treatment discontinuation or end of the follow-up period (whichever came first)

^b^Costs of capitation patients were replaced with fee-for-service proxy; all costs were adjusted by consumer price index

^c^Biologic therapy refers to the following anti-TNFα agents: etanercept, adalimumab, infliximab, and golimumab

^d^Medical cost = hospitalization cost + ER cost + office visit cost. Number of months is defined as the rounding of number of days on treatment divided by 30. If number of months equals to 0, then it is assigned as 1

^e^Pharmacy cost = Biologic treatment cost + DMARD treatment cost

*DMARD* disease-modifying antirheumatic drug, *ER* emergency room, *PsA* psoriatic arthritis, *PPPM* per patient per month, *SD* standard deviation, *TNFα* tumor necrosis factor-α

## Discussion

In this descriptive claims-based study, treatment patterns differed among PsA patients who remained on their first-line of anti-TNFα biologic therapy compared with those who switched to additional lines of anti-TNFα biologic therapy. PsA patients who remained on their first-line anti-TNFα biologic therapy showed longer persistence and fewer treatment modifications of the first-line therapy compared to those who switched to a second-, third- or greater line of therapy. Time to first-line treatment modification was longer for patients who switched to third- or greater lines of therapy than for those who did not switch or switched to second-line. Time-to-switch and time to first-line modification was progressively shorter with each line of therapy for patients who received third- or greater line. PPPM medical costs were greater for patients who did not switch ($322) than for patients who switched to a second- ($167) or third- or greater line ($217) of therapy. PPPM pharmacy costs were higher than medical costs, and were greater for patients with three or more lines of anti-TNFα biologic therapy ($2539) compared with those with only a first- ($1985) or a second-line ($2045) of therapy.

Similar to other US studies, our study found that etanercept and adalimumab were the most common index anti-TNFα biologic therapies [31–35]. We also found that these two drugs were the most frequent anti-TNFα biologic agents used in patients who switched to other lines of therapy. Reasons for differences in treatment patterns across lines of therapy groups are not clear, but may be influenced by dissimilarities in response to anti-TNFα biologic therapy, comorbidities, health insurance, change of physician, adverse events (AEs), disease characteristics, disease severity, or patient preference [6, 24, 27, 34, 36, 37]. Gender may also be another factor that has influenced switching [34]. As we observed, a lower percentage of females persisted on their first-line therapy compared with those who switched to either a second- or third- or greater line of anti-TNFα biologic treatment. In addition, several non-US studies found that women with PsA have shorter anti-TNFα biologic drug survival than men [52–54]. The increase in switching and the rate of modification with each line of therapy observed in patients who switched to three or more lines of therapy may indicate a lack of treatment response, poor tolerance, and/or high disease severity in these patients. In support of this, one prior non-US study found that lack of treatment effect and AEs were the main reasons why patients with PsA switched their anti-TNFα biologic treatment [52].

Our findings are comparable to that of other US studies which found that the rate of switching anti-TNFα biologic treatment over 1 year is low in PsA patients (range 2.8 to 25 %) [31–35]. Non-US studies have also found that only a minority of PsA patients switch anti-TNFα biologic therapy within 1 year [54–56]. In previous US studies, patients who did not switch either remained on their index anti-TNFα biologic, restarted their index therapy after a treatment gap, or discontinued therapy [31–35]. We did not evaluate the rate of restarting or discontinuation of therapy.

Similar to the US study in 2014 by Zhang et al. [31], we found that, for the overall study population, the rate of treatment modification was low, with the most common modifications being the addition or removal of a DMARD. Zhang et al. found that 7 % of PsA patients added a nonbiologic DMARD to their index anti-TNFα biologic therapy [31]. Our findings expand on those of Zhang et al., who did not assess treatment patterns associated with multiple lines of therapy. In our study, modification of first-line therapy was more common in patients with multiple lines of therapy compared with those who did not switch. We found that patients who had three or more lines of therapy added a DMARD to their first-line anti-TNFα biologic therapy at a higher rate (18.9 %) compared with those who did not switch (8.4 %) or who switched to second-line therapy (11.1 %) over the 3-year follow-up period. We also found that about 14 % of patients that switched to second-line TNFα biologic therapy added a DMARD to their second-line anti-TNFα biologic therapy. In contrast, no patients who switched to three or more lines of therapy added a DMARD to their second-line therapy; instead, 22 % removed a DMARD. Currently, it is unclear what factors influence the rate and type of anti-TNFα biologic treatment modification.

Previous US studies assessed the direct healthcare costs of anti-TNFα biologic therapy [35, 38–40]. They also did not evaluate the influence of switching on drug and healthcare costs [35, 38–40]. We found monthly medical costs were highest for patients who did not switch and lowest for those that switched to two lines of therapy over the 3-year follow-up period. In patients who switched to three or more lines of anti-TNFα biologic therapy, first-line therapy was associated with higher medical costs than second- or third- or greater lines of treatment, which had similar associated medical costs. Monthly pharmacy costs were higher in patients who switched compared with those who did not and highest in those that switched to at least three lines of treatment. The higher pharmacy costs with switching may indicate a need for additional medication possibly due to dose escalation, disease progression, or the presence of comorbidities in these patients. The cost of DMARDs compared with anti-TNFα biologic therapy was not differentiated in our analysis; hence, it is not clear how these therapies influenced overall pharmacy costs.

Although several prior US studies have evaluated the cost of anti-TNFα biologic treatment in PsA [32, 35, 38–40], our study is the first to assess costs associated with switching to multiple lines of anti-TNFα biologic therapy. Direct comparison between our study and prior studies is difficult due to methodological differences in determining costs. However, in general across the studies, the annual cost for first-line therapy with anti-TNFα biologic treatment per patient ranged from about $17,000 to $29,000 [32, 35, 39, 40], which is similar to the annual pharmacy cost for first-line treatment of about $24,000 seen in our study.

The current study is a retrospective observational analysis of administrative claims databases with descriptive findings only. In addition, we used claims data that did not capture the reasons for switching. Therefore, we do not know how treatment response, physician beliefs, tolerability, efficacy, treatment modification, or treatment discontinuation influenced switching. This study was limited to PsA patients with commercial health coverage of Medicare. Consequently, it only evaluated PsA patients during the 3-year study period and it is unclear if the findings are translatable to those that are uninsured or on Medicaid. In addition, the limited sample size in the latter lines of therapy limit our ability to generalize our findings to a larger PsA population. Our study is also limited by that fact we only evaluated continuous users. We also excluded patients who ceased therapy for >60 days; therefore, we did not capture patients who stopped anti-TNFα biologic treatment and subsequently restarted with the same or different treatment. Finally, diagnoses on claims may be coded incorrectly or not coded at all, thereby potentially introducing measurement error with respect to ICD-9 based variables. We also did not take into consideration the potential effect of rebates, discounts, or other price concessions.

## Conclusion

This descriptive claims-based study found PsA patient treatment patterns and healthcare costs differed depending on the number of times a patient switched their anti-TNFα biologic therapy. Most PsA patients remained on their first-line of therapy over the 3-year study period. Overall, the rate of treatment modification was low with patients who did not switch treatment having the lowest rate of modification. Time to switch and time to first treatment modification was progressively shorter for patients who received at least three lines of therapy—potentially an indication of reduced response. The highest PPPM medical costs were in patients that did not switch and the highest PPPM pharmacy costs occurred in patients with three or more lines of anti-TNFα biologic therapy. The findings of this give healthcare providers a better understanding of the real-world treatment pattern and economic impact of PsA.

## Abbreviations

AAD, American Academy of Dermatology; anti-TNFα, anti-tumor necrosis factor alpha; AS, Ankylosing spondylitis; DMARD, Disease-modifying antirheumatic drug; ER, Emergency room; EULAR, European League Against Rheumatism; FFS, Fee-for-service; GRAPPA, Group for Research and Assessment of Psoriasis and Psoriatic Arthritis; HMOs, Health maintenance organizations; ICD-9-CM, International Classification of Diseases, Ninth Revision, Clinical Modification; MTX, Methotrexate; N/A, Not available; NSAIDs, Non-steroidal anti-inflammatory drugs; POS, Point of service; PPPM, Per patient per month; PsA, Psoriatic arthritis; RA, Rheumatoid arthritis; SD, Standard deviation; US, United States
